# Efficacy of Recent Emissions Controls on Road Vehicles in Europe and Implications for Public Health

**DOI:** 10.1038/s41598-017-01135-2

**Published:** 2017-04-25

**Authors:** Roy M. Harrison, David C. Beddows

**Affiliations:** 1 School of Geography, Earth and Environmental Sciences, University of Birmingham, Edgbaston, Birmingham B15 2TT UK; 2 Department of Environmental Sciences/Center of Excellence in Environmental Studies, King Abdulaziz University, PO Box 80203, Jeddah, 21589 Saudi Arabia

## Abstract

Road traffic is a major source of urban air pollution responsible for substantial premature mortality. Until recently, attention has focussed primarily on exhaust emissions of particulate matter from traffic as a causal factor. From analysis of air quality measurement data from the UK and France, we demonstrate that road traffic exhaust has a far greater impact on concentrations of nitrogen dioxide than of PM_2.5_. PM_2.5_ and carbonaceous particle concentrations have been declining appreciably since 2010/11 due to the use of diesel particle filters, but little change is seen in nitrogen dioxide over the period from 1995 to 2015. It is shown that the effect of NO_2_ from road traffic upon premature mortality was ten-fold greater than that of PM_2.5_ even before the widespread use of diesel particle filters, and is now considerably larger. The overwhelming contribution of diesel compared to gasoline-fuelled vehicles to emissions of both PM_2.5_ and NO_2_ emphasises the importance of further controls on emissions from diesels.

## Introduction

The adverse effects of air pollution exposure on human health are well established^1–5^, and the recent Volkswagen emissions scandal has highlighted the contribution of engineering controls in the achievement of improved air quality. Catalytic converters fitted to gasoline-powered vehicles have led to huge reductions in airborne concentrations of carbon monoxide and benzene in cities throughout the developed world^6, 7^, but despite their well demonstrated adverse effects on human health, particulate matter^8, 9^ and nitrogen dioxide^10, 11^ have yet to be adequately controlled.

In Europe, vehicle emissions are regulated through the Euro standards, and the Euro 5b standard, introduced in September 2011 for light duty vehicles, and Euro VI introduced in January 2013 for heavy duty vehicles, placed limits upon not only the mass of particles emitted per kilometre, but also regulated the particle number^12^ which has required the fitting of diesel particle filters. In the case of oxides of nitrogen, there has been a gradual tightening of the type approval standard, from 0.5 g km^−1^ for Euro 3 light duty vehicles (in 2000) to 0.18 g km^−1^ for Euro 5a in September 2009 and a further reduction to 0.08 g km^−1^ for Euro 6 introduced in September 2014. In the case of heavy duty vehicles, Euro III required 5 g kWh^−1^ for NO_x_ in October 2000, reducing to 2.0 g kWh^−1^ in October 2008 and to 0.4 g kWh^−1^ with Euro VI in January 2013. As highlighted by the Volkswagen emissions scandal, the type approval standards set by the European Union may not be representative of real-world driving emissions and various studies have shown a huge discrepancy between on-the-road emissions and the type approval limits tested in the laboratory^13, 14^.

The only truly representative way in which to evaluate the efficacy of emission controls is through ambient air measurements. Font and Fuller^15^ link changes in air quality at London sites to emission trends, but typically pollutants have multiple sources, and consequently changes in ambient concentrations cannot easily be attributed to an individual source category. In the case of road traffic, this problem can be overcome through the use of paired site studies in which a heavily trafficked roadside air sampling site is paired with a background monitoring site, the difference between the two representing the road traffic increment caused by emissions on the road in question^16^. In this study, we have used routine ambient air quality measurements downloaded from the UK Automatic Urban and Rural Network^17^ and Airparif^18^ for such paired sites in London, Glasgow and Paris in order to evaluate the change in vehicle emissions over recent years since the introduction of diesel particle filters and the implementation of the stricter Euro regulation for NO_x_ emissions.

## Methods and Results

**Figure 1:**
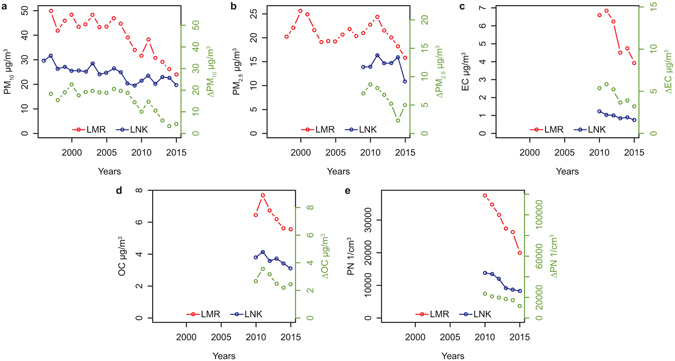
Annual mean pollutant concentration trends at Marylebone Road and North Kensington and the road traffic increment (Δ). (**a**) PM_10_; (**b**) PM_2.5_; (**c**) Elemental Carbon; (**d**) Organic Carbon and (**e**) Particle Number Count.

The paired sites of London Marylebone Road (roadside) and London North Kensington (urban background) have been used extensively in the past for evaluation of road traffic incremental pollution^19, 20^. Traffic flows, and the vehicle mix have not changed appreciably over the period of the measurements (see Supplementary Fig. S1). The trends in annual mean concentrations of particulate matter mass expressed as PM_10_ and PM_2.5_, particle number, elemental carbon (EC) and organic carbon (OC) appear in Fig. 1, and Supplementary Table S1. The reduction in roadside incremental PM_10_ mass between 2009–11 (average) and 2015 is 8.7 µg m^−3^ (66.6%), and for PM_2.5_ is 3.0 µg m^−3^ (37.5%). Since exhaust emissions of particulate matter are made up predominantly from EC and organic matter, an approximate mass can be estimated from the sum of EC + 1.2 OC^21^. Data are available only since 2010 and the difference between 2010–11 and 2015 for EC is 2.44 µg m^−3^ (43.4% decrease) and for 1.2 OC is 0.80 µg m^−3^ giving a total reduction in particulate matter mass of 3.2 µg m^−3^ (35.5% decrease) which is very close to that for PM_2.5_ (3.0 µg m^−3^; 37.5% decrease) giving a clear indication that this is predominantly responsible for the reduction of the fine particle mass over this period.

The trends in particle number count show a reduction in the roadside increment from 22549 cm^−3^ in 2010–11 to 11657 cm^−3^ in 2015, an overall percentage reduction of 48.3%. The particle number count is dominated by the ultrafine particle fraction whose emission is sensitive to the sulphur content of motor fuel^22^. As fuel sulphur levels have been fairly stable over the period of these measurements, the reduction in particle number count would appear to be attributable to the introduction of DPFs which gives some reassurance the reduction in particle mass was accompanied by a commensurate reduction in the concentration of ultrafine particles.

**Figure 2:**
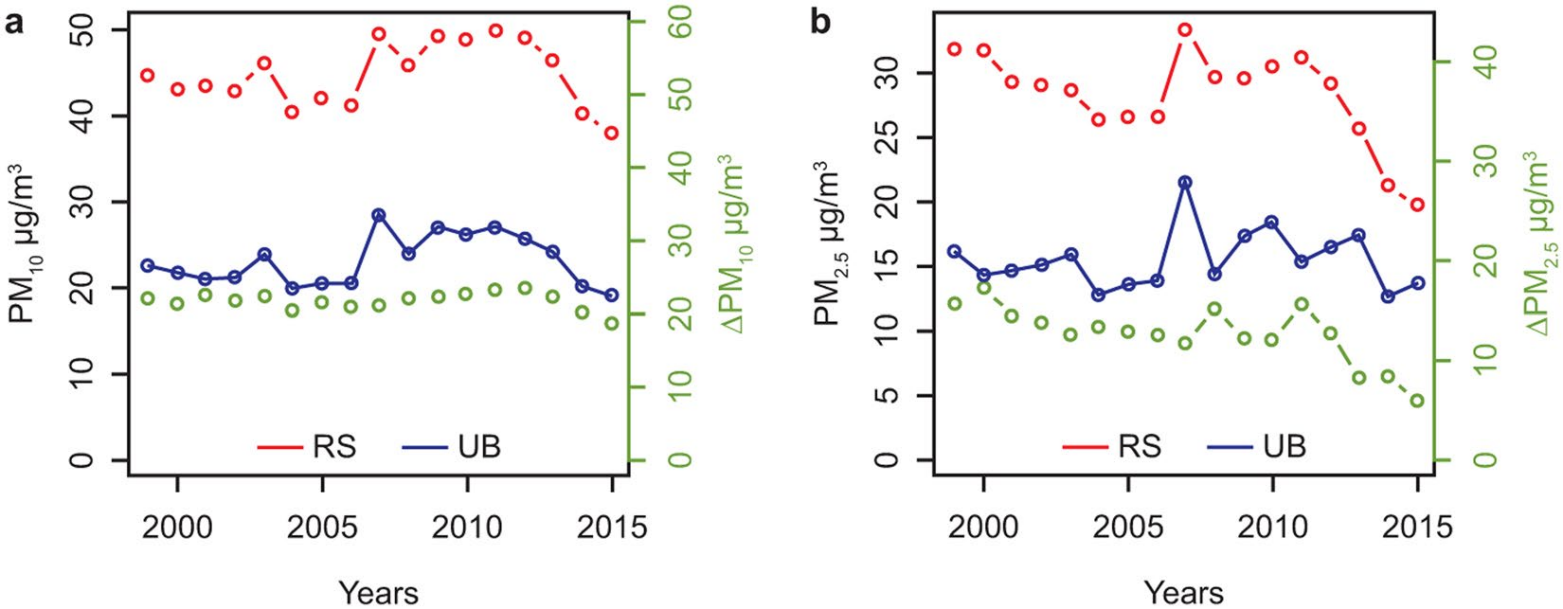
Annual mean concentrations of particulate matter measured in Paris at Vitry-Sur-Seine (UB) and Boulevard Périphérique Auteuil (RS) and road traffic increment (Δ). (**a**) PM_10_ and (**b**) PM_2.5_.

The only other paired sites with suitable data in the UK are in Glasgow. In Supplementary Fig. S3, data from the Glasgow roadside site are plotted alongside those from the Glasgow background sites, Glasgow Central (2000–2012) and Glasgow, Townhead (2013–2015). This shows little change in incremental NO, NO_2_ and NO_x_ for the entire period, but a substantial recent fall in ΔPM_2.5_ and a possible upward trend in ΔPM_10_. Further data on particulate matter mass are available from paired sites in Paris, Boulevard Périphérique Auteuil and Vitry-Sur-Seine, separated by 12.5 km. In this case, taking the same time period of 2009–11 to 2015, PM_10_ reduced in concentration by 4.0 µg m^−3^ (17.5%) and PM_2.5_ by 7.3 µg m^−3^ (55.1%) (Fig. 2). In this case an increase in coarse particle mass is implied.

**Figure 3:**
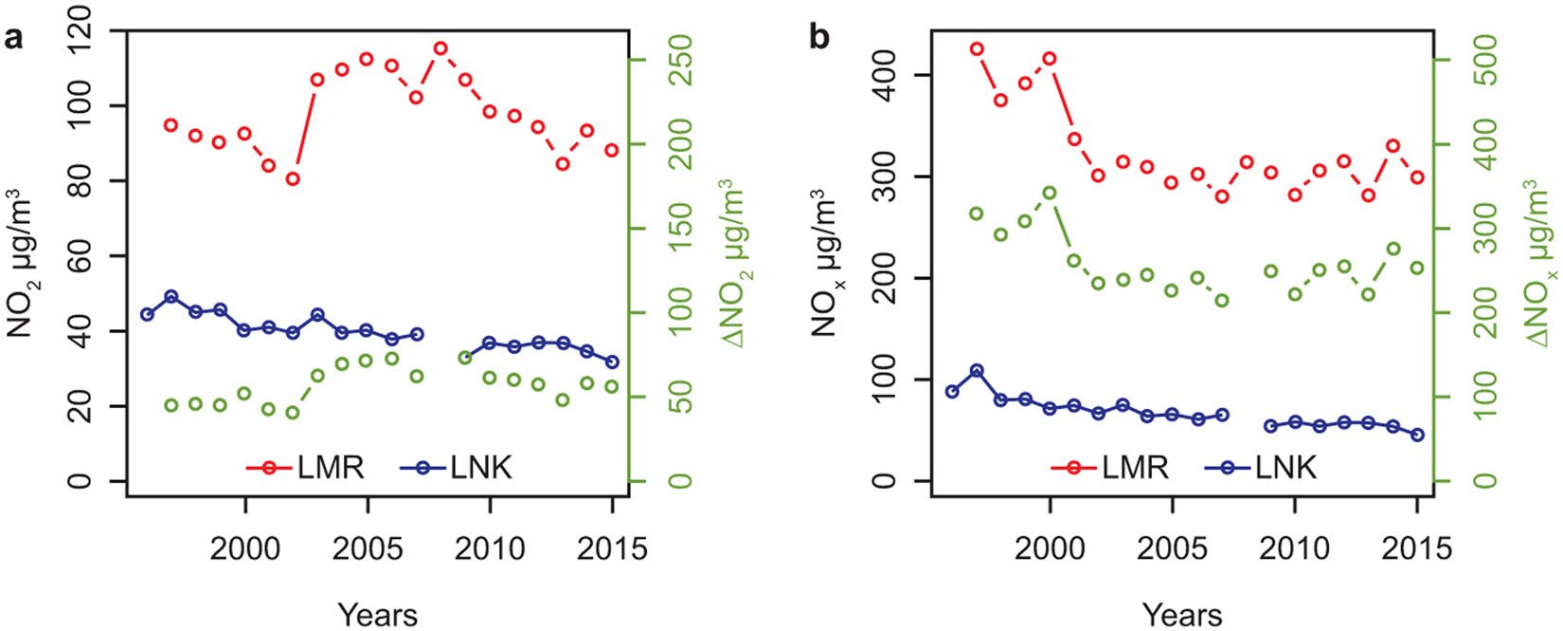
Trend in annual mean concentrations of oxides of nitrogen measured in London at North (LNK) Kensington and Marylebone Road (LMR), and road traffic increment (Δ) (**a**) NO_2_ and (**b**) NO_x_.

Regarding the gas-phase pollutants, the reduction in carbon monoxide and in benzene occurred much earlier as a result of the introduction of catalytic converters on gasoline vehicles. The trend since 1995 is shown in Supplementary Fig. S2 for comparison. Trends in incremental NO_x_ and NO_2_ for Marylebone Road appear in Fig. 3 and extend back to 1997. These show no obvious improvement in concentrations of NO_x_ between 2001 and 2015, while concentrations of nitrogen dioxide have increased slightly over this period, having gone through a maximum in 2008.

## Discussion and Implications

Our inference from these results is that the introduction of diesel particle filters (DPF), required since 2011, is already having a very significant benefit for air quality through a reduction of around 36% in vehicle exhaust emissions of PM_2.5_ in London since 2009–11. This improvement has also been seen in the emissions of ultrafine particles from road traffic which is the major source of this pollutant. On the other hand, concentrations of oxides of nitrogen do not appear to have benefitted from a reduction in the type approval standard for emissions of 84% since the introduction of Euro 3 in 2000 for light duty vehicles, and 80% since 2000 for heavy duty vehicles.

In 2010/11 before the introduction of diesel particle filters, the incremental concentration of PM_2.5_ at Marylebone Road was 8.7 µg m^−3^, for nitrogen dioxide was 61.6 µg m^−3^, and for NO_x_ was 222 µg m^−3^. As that NO_x_ disperses in the urban atmosphere, the NO, which predominates in the street canyon, is largely converted by oxidation to NO_2_ and the NO_2_/NO_x_ ratio at North Kensington in 2010/11 was 36.4/56.5 = 0.64. Consequently, the incremental NO_x_ has an equivalent concentration of 222 × 0.64 = 143 µg m^−3^ of NO_2_ at a background urban atmospheric NO_2_/NO_x_ ratio. This implies that in 2010/11 the ratio of contributions from traffic to background urban concentrations of NO_2_/PM_2.5_ was 143/8.7 or approximately 16.4 in the background urban atmosphere where most human exposure occurs

The relative public health impacts of PM_2.5_ and NO_2_ can be judged crudely from their effect on all-cause mortality. This is not straightforward as PM_2.5_ and NO_2_ concentrations are normally highly correlated and under such circumstances the results of two pollutant models carry substantial uncertainty in the attribution of health effects to each pollutant. The WHO HRAPIE project^3^ recommended a Hazard Ratio coefficient for PM_2.5_ of 1.062 per 10 µg m^−3^. HRAPIE^3^ also recommend a coefficient linking NO_2_ and all-cause mortality of 1.055 per 10 µg m^−3^ but recommended reducing this by up to 33% to allow for double counting of effects of particulate matter, due to its high correlation with NO_2_. Subtracting the maximum of 33% from the NO_2_ coefficient yields a Hazard Ratio of 1.037. HRAPIE recommended application of the coefficient for concentrations >20 µg m^−3^. However, new evidence and in particular that from the ESCAPE study^23^ has shown linearity to much lower concentrations (the lowest concentration of NO_2_ in the ESCAPE cohorts was 1.5 µg m^−3^) and we therefore feel confident in applying this coefficient to the annual mean concentrations in London, which in any case well exceed 20 µg m^−3^. HRAPIE did not recommend attenuation of the PM_2.5_ coefficient for the effects of NO_2_, although in some studies in the meta-analysis upon which it is based, NO_2_ may also have exerted an effect. They did however note the possibility of double counting. HRAPIE^3^ noted that studies with a better quality of exposure estimate yielded on average higher hazard ratios and suggested that the result of the meta-analysis which they used (Relative risk of 1.062, with 95% CI = 1.040–1.083) could result in an underestimation of the exposure-response slope. This being the case, we have chosen not to attenuate the coefficient for PM_2.5_. Had we done so, the relative proportion of health impact attributed to nitrogen dioxide would have been greater. This then gives a ratio of impact per unit mass for NO_2_/PM_2.5_ of 0.037/0.062 = 0.60. However, if the relative impacts of emission of the two pollutants from road traffic is to be estimated, the relative concentrations of the two pollutants in the background urban atmosphere (16.4 in 2010/11 and 35 in 2015) need to be taken into account, giving a ratio of 9.8 in 2010/11 and 21 in 2015 for the mortality due to traffic emitted nitrogen dioxide relative to PM_2.5_ from traffic. The implication is that the premature mortality impact of nitrogen dioxide from road traffic in 2010/11 exceeded that due to PM_2.5_ from this source by almost ten-fold and in 2015 by more than twenty-fold. There are uncertainties around this ratio. The specificity of the effects which correlate with NO_2_ concentrations has not been firmly established, and there may be a contribution from other, highly correlated pollutants such as ultrafine particles. On the other hand, the coefficient used for PM_2.5_ has not been attenuated to allow for the effect of NO_2_, and may over-estimate the impacts of PM_2.5_.

Table 1 shows a breakdown for 2014 of the emissions of PM_2.5_ and NO_x_ taken from the National Atmospheric Emissions Inventory^24^ disaggregated according to the vehicle type and fuel. This shows that diesel vehicles in urban areas are responsible for 97% of the PM_2.5_ exhaust emissions from road traffic and 90% of the NO_x_ emissions. In the case of NO_x_, which arguably is responsible for a larger burden of disease than the PM_2.5_ from road traffic, there is a clear and urgent need to reduce the volume of diesel traffic on urban roads or to ensure that new vehicles entering the road emit far less NO_x_ per kilometre than the current diesel fleet. The National Atmospheric Emissions Inventory^24^ predicts a reduction in NO_x_ from road transport from 300 kt in 2014 to 174 kt in 2020 and 128 kt in 2025. It remains to be seen whether such reductions will in fact be achieved as this will depend critically upon the ability of motor manufacturers to install effective abatement devices on the vehicles which they produce, and that those devices remain fully effective over the lifetime of the vehicle. Past history gives little confidence that this will be achieved.

**Table 1:** Exhaust emissions of PM_2.5_ and NO_x_ from road traffic in UK urban areas in 2014 (kt) (from ref. 24).

	PM_2.5_		NO_x_ (as NO_2_)	
	Gasoline	Diesel	Gasoline	Diesel
Passenger cars	0.036	0.87	9.9	44
Light goods vehicles	0.001	0.49	0.4	20
Heavy goods vehicles and buses	—	0.40	—	34.8
Mopeds and motorcycles	0.022	—	0.3	—
Total	0.059	1.76	10.6	94.8

## Electronic supplementary material

Supplementary Information
