# Supplementary material for: Efficacy of Recent Emissions Controls on Road Vehicles in Europe and Implications for Public Health
Source: Sci Rep. 2017 Apr 25;7:1152. doi: 10.1038/s41598-017-01135-2 (PMC5430659; doi:10.1038/s41598-017-01135-2)
Supplement: Supplementary file 1 — Supplementary Information [file 41598_2017_1135_MOESM1_ESM.pdf]

SUPPLEMENTARY INFORMATION

Efficacy of Recent Emissions Controls on Road Vehicles in Europe and Implications for Public Health, Roy M. Harrison and David C. Beddows

Projections of the National Atmospheric Emissions Inventory (NAEI)

The National Atmospheric Emissions Inventory [24] indicates a reduction of $PM_{2.5}$ from exhaust emissions between 2010 and 2014 of 42%. If, however, the data for urban emissions alone are considered, the exhaust emissions of $PM_{2.5}$ reduce from 2.80 to 1.85 kt, a reduction of 34%. When the emissions of $PM_{2.5}$ from tyre and brake wear and road surface abrasion are included, the $PM_{2.5}$ emissions in 2010 of 6.03 kt reduce to 5.12 kt in 2014, a reduction of 15%. These data suggest that the percentage reduction in the incremental sum of elemental carbon and organic matter (35.5%) probably reflects quite well the reduction in exhaust emissions while the reduction in total measured $PM_{2.5}$ well exceeds the estimate from the NAEI, even allowing for the shorter period covered by the NAEI data.

Details of the London Sampling Sites

The Marylebone Road site is located within a street canyon in central London and three lanes of heavy traffic in each direction with an overall annual vehicle count of 77,500 ± 6000 (s.d.) (over 2000-2015) vehicles per day causes a substantial increment in pollutant concentrations. Over the 2000-2015 period the annual average daily traffic flow back and forth along the Marylebone Road has remained fairly constant and the percentages of HGV, LGV, cars and taxis and motorbikes have not changed substantially and in 2015 were 3.9%, 12.2%, 74.8% and 5.9% of the total respectively (see Fig. 1) (www.dft.gov.uk/traffic-counts/cp.php). The London North Kensington site, which is located some 4.1 km to the West is widely taken as representative of background air quality in central London [25] and provides a suitable background for subtraction.

Details of the Paris Sampling Sites

Boulevard Périphérique Auteuil

Roadside site located on the Paris ring road 80kph limit.

240,000 vehicles/day, 8 lane highway.

Bd Péripherique - Porte d Auteuil

75016, PARIS 16ème

Vitry-Sur-Seine

103 rue Paul Armangot

94400, VITRY-SUR-SEINE

Background site within a residential area in the south of Paris and ~2.5km beyond ringroad, 500m East of the D7 highway and 2km East of the A6B highway.

Details of the Glasgow Sites

Glasgow Central

Lat/long 55.859170, -4.258889

Located on the pavement of Hope Street adjacent to Glasgow Central Station. The nearest road is subject to frequent congestion during peak traffic flow periods. Street canyon, one way traffic 30mph limit, two lanes, one of which is used as a bus lane.

Glasgow, Townhead

Latitude/Longitude: 55.865782, -4.243631

The monitoring station is located inside a residential area, which lies in the perimeter established by St Mungo's Avenue and Kennedy Street, in Glasgow's city centre. The surrounding area comprises residential houses, gardens and some businesses, in an urban

environment. The M8 motorway is situated approximately 360 m to the North, the A803 to the East and A804 to the West.

Changes in PM_{10} and Coarse ($PM_{2.5-10}$) Particle Mass Increment

In the London data (Figure 1) there is a decline in both the PM_{10} and $PM_{2.5-10}$ increment between the sites. The fact that the coarse particle mass ($PM_{2.5-10}$) declines over this period requires explanation. Vehicle exhaust particulate matter is generally considered to be almost entirely in the $PM_{2.5}$ size range, while non-exhaust emissions are predominantly coarse but also make a contribution to $PM_{2.5}$. There appears to be a reduction in the non-exhaust emissions, which arise from brake wear, tyre wear and road surface wear. Such emissions have in the past been linked closely to the nature and behaviour of heavy duty vehicles on Marylebone Road, [26,27] and the explanation for this temporal change may well relate to changes in the bus fleet over this period.

In the data from Paris (Figure 2), PM_{10} declines very little between 2011 and 2015 compared to $PM_{2.5}$ and there is an increase in $PM_{2.5-10}$. This seems likely to have arisen from increased non-exhaust particle emissions (brake wear, tyre wear and resuspension) which are predominantly in this size range. Changes in the volume or mix of traffic may have contributed, but traffic data were not available to investigate this.

In the case of Glasgow (Figure S3), the fall in $PM_{2.5}$ from 2010 to 2015 far exceeds that for PM_{10}. The PM_{10} data at the kerbside site show large inter-annual variations for which there is no ready explanation. Both the kerbside and background sites show a general decline in PM_{10} after 2006, but the kerbside increment remains relatively constant from 2012 to 2014, implying an increase in $PM_{2.5-10}$, as for the Paris sites.

The one consistent feature at all of the sites is the decline in the $PM_{2.5}$ kerbside increment after 2010. Coarse particles have a shorter atmospheric lifetime and are inherently less predictable and may also be affected by changes in the vehicle fleet as suggested for London.

Measurement Methods

All sites belong to a national or regional network and are subject to rigorous quality assurance. PM_{10} and $PM_{2.5}$ are reported as hourly averages from all sites, measured with a TEOM-FDMS which has demonstrated equivalence with the European Reference Method according to EN12341:2014. Nitrogen dioxide is measured using chemiluminescence analysers which have demonstrated compliance with the CEN reference method set out in EN14211:2012.

REFERENCES

24. National Atmospheric Emissions Inventory. http://naei.defra.gov.uk/ (last updated 05/10/2016).

25. Bigi, A. & Harrison, R. M. Analysis of the air pollution climate at a central urban background site. *Atmos. Environ.* **44**, 2004-2012 (2010).

26. Thorpe, A. J., Harrison, R. M., Boulter, P. G. & McCrae, I. S. Estimation of particle resuspension source strength on a major London road. *Atmos. Environ.* **41**, 8007-8020 (2007).

27. Carslaw, D. C., Ropkins, K. & Bell, M. C. Change-point detection of gaseous and particulate traffic-related pollutants at a roadside location. *Environ. Sci. Technol.*, **40**, 6912-6918 (2006).

TABLE LEGEND

Table S1. Annual mean concentrations of the pollutants at London North Kensington (LNK) and London Marylebone Road (LMR). All concentrations in µg m^{-3}. Downloaded from http://uk-air.defra.gov.uk/

FIGURE LEGENDS

Fig. S1. Average yearly daily traffic flows of various vehicle categories expressed as a percentage of the total traffic counts.

Fig. S2. Trend in annual mean concentrations of pollutants measured in London and the traffic increment (Δ). (a) Carbon monoxide measured at Marylebone Road (LMR) and North Kensington (LNK); (b) benzene measured at Marylebone Road (LMR) and Eltham (LME).

Fig. S3. Annual mean concentrations of particulate matter and oxides of nitrogen at the paired kerbside and urban background sites in Glasgow, and the traffic increment (Δ). (a) NO; (b) NO_2; (c) NO_x; (d) CO; (e) $PM_{2.5}$; (f) PM_{10}.

Table S1. Annual mean concentrations of the pollutants at London North Kensington (LNK) and London Marylebone Road (LMR). All concentrations in µg m^{-3}. Downloaded from http://uk-air.defra.gov.uk/

				LNK			
	NO2	*NOx*	*CO*	*PM10*	*PM2.5*	*EC*	*OC*
2009	33.34	54.63	0.29	19.57	13.87		
2010	36.83	58.65	0.26	21.40	14.00	1.22	3.79
2011	36.12	53.73	0.22	23.72	16.29	1.03	4.16
2012	36.72	57.43	0.27	20.23	14.68	1.02	3.57
2013	36.85	57.91	0.25	23.03	14.72	0.85	3.74
2014	34.55	53.36	0.29	22.66	15.93	0.89	3.44
2015	31.69	45.54	0.32	19.71	10.86	0.75	3.12

				LMR			
	NO2	*NOx*	*CO*	*PM10*	*PM2.5*	*EC*	*OC*
2009	107.05	303.25	0.7	34.03	20.98		
2010	98.47	281.18	0.65	31.61	22.67	6.61	6.47
2011	97.18	305.64	0.66	38.35	24.39	6.88	7.72
2012	94.27	313.98	0.59	30.75	21.51	6.26	6.73
2013	84.66	280.82	0.51	29.17	20.03	4.52	6.21
2014	93.54	328.79	0.52	26.30	18.20	4.78	5.62
2015	88.28	298.42	0.51	24.09	15.84	3.94	5.57

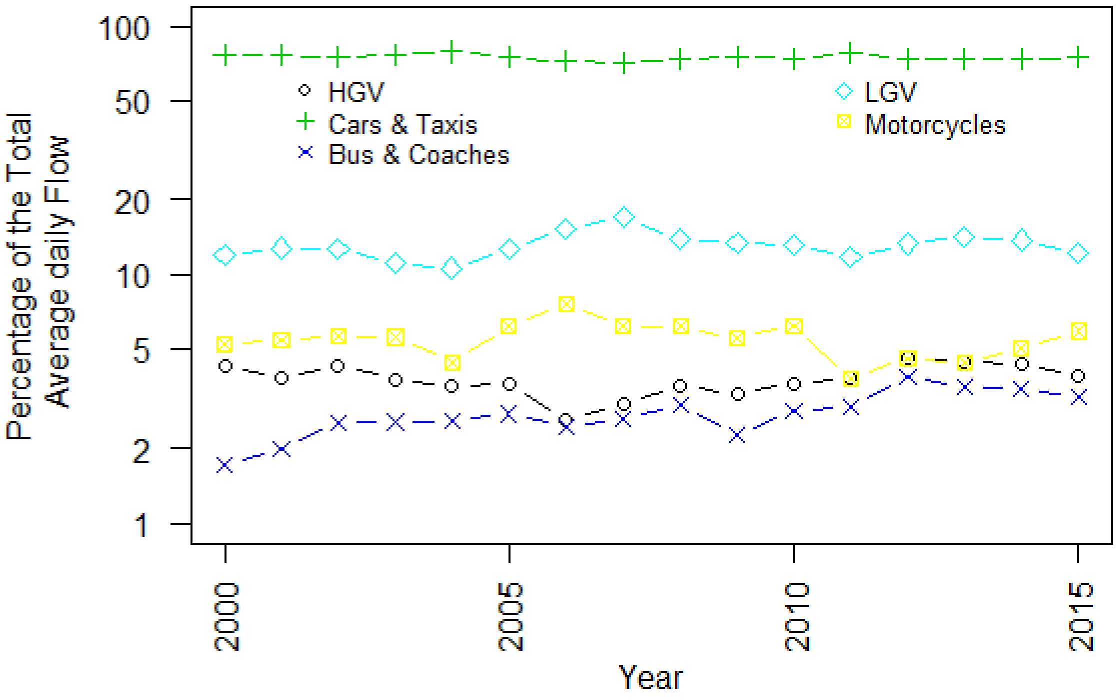

Fig. S1. Average yearly daily traffic flows of various vehicle categories expressed as a percentage of the total traffic counts.

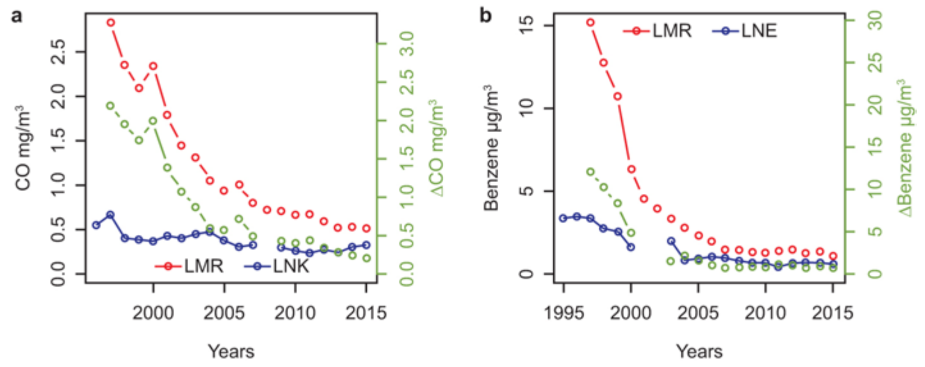

Fig. S2. Trend in annual mean concentrations of pollutants measured in London and the traffic increment (Δ). (a) Carbon monoxide measured at Marylebone Road (LMR) and North Kensington (LNK); (b) benzene measured at Marylebone Road (LMR) and Eltham (LME).

.

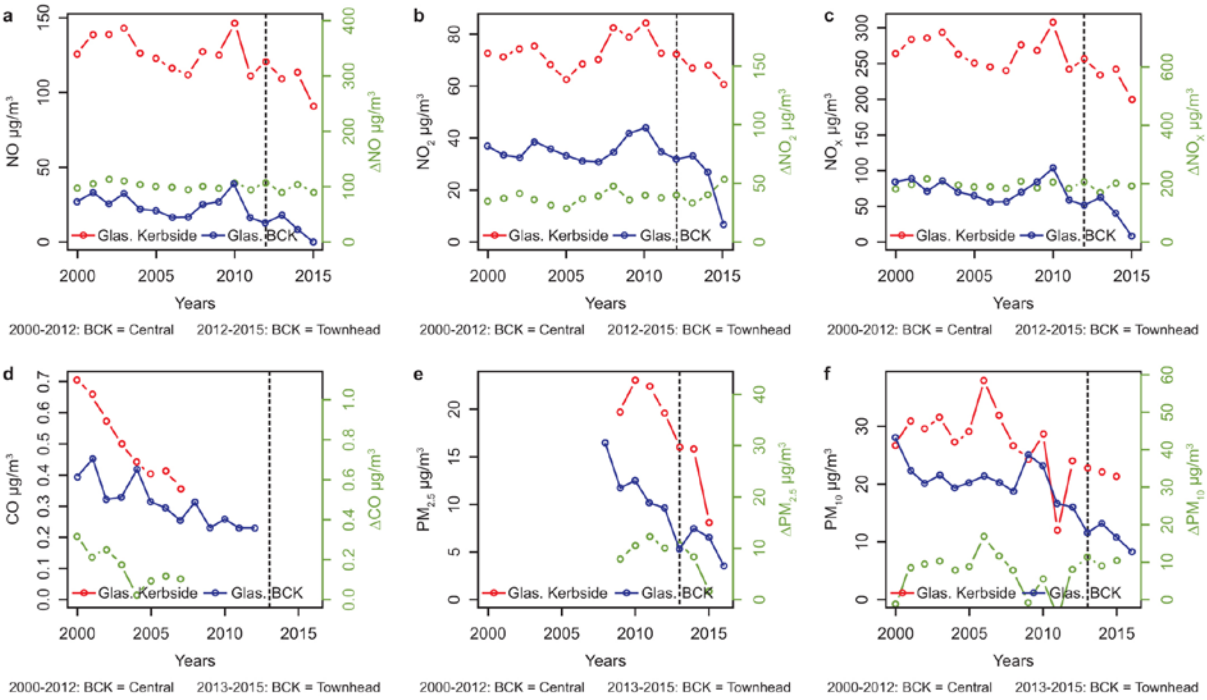

Fig. S3. Annual mean concentrations of particulate matter and oxides of nitrogen at the paired kerbside and urban background sites in Glasgow, and the traffic increment (Δ). (a) NO; (b) NO_2; (c) NO_x; (d) CO; (e) $PM_{2.5}$; (f) PM_{10}.
